# A Novel Composite Indicator of Predicting Mortality Risk for Heart Failure Patients With Diabetes Admitted to Intensive Care Unit Based on Machine Learning

**DOI:** 10.3389/fendo.2022.917838

**Published:** 2022-06-29

**Authors:** Boshen Yang, Yuankang Zhu, Xia Lu, Chengxing Shen

**Affiliations:** ^1^ Department of Cardiology, Shanghai Jiao Tong University Affiliated Sixth People’s Hospital, Shanghai, China; ^2^ Department of Gerontology, Xinhua Hospital affiliated to Shanghai Jiaotong University School of Medicine, Shanghai, China

**Keywords:** heart failure, diabetes, machine learning, hospital mortality, indicator

## Abstract

**Background:**

Patients with heart failure (HF) with diabetes may face a poorer prognosis and higher mortality than patients with either disease alone, especially for those in intensive care unit. So far, there is no precise mortality risk prediction indicator for this kind of patient.

**Method:**

Two high-quality critically ill databases, the Medical Information Mart for Intensive Care IV (MIMIC-IV) database and the Telehealth Intensive Care Unit (eICU) Collaborative Research Database (eICU-CRD) Collaborative Research Database, were used for study participants’ screening as well as internal and external validation. Nine machine learning models were compared, and the best one was selected to define indicators associated with hospital mortality for patients with HF with diabetes. Existing attributes most related to hospital mortality were identified using a visualization method developed for machine learning, namely, Shapley Additive Explanations (SHAP) method. A new composite indicator ASL was established using logistics regression for patients with HF with diabetes based on major existing indicators. Then, the new index was compared with existing indicators to confirm its discrimination ability and clinical value using the receiver operating characteristic (ROC) curve, decision curve, and calibration curve.

**Results:**

The random forest model outperformed among nine models with the area under the ROC curve (AUC) = 0.92 after hyper-parameter optimization. By using this model, the top 20 attributes associated with hospital mortality in these patients were identified among all the attributes based on SHAP method. Acute Physiology Score (APS) III, Sepsis-related Organ Failure Assessment (SOFA), and Max lactate were selected as major attributes related to mortality risk, and a new composite indicator was developed by combining these three indicators, which was named as ASL. Both in the initial and external cohort, the new indicator, ASL, had greater risk discrimination ability with AUC higher than 0.80 in both low- and high-risk groups compared with existing attributes. The decision curve and calibration curve indicated that this indicator also had a respectable clinical value compared with APS III and SOFA. In addition, this indicator had a good risk stratification ability when the patients were divided into three risk levels.

**Conclusion:**

A new composite indicator for predicting mortality risk in patients with HF with diabetes admitted to intensive care unit was developed on the basis of attributes identified by the random forest model. Compared with existing attributes such as APS III and SOFA, the new indicator had better discrimination ability and clinical value, which had potential value in reducing the mortality risk of these patients.

## Introduction

Heart failure (HF) is the end-stage manifestation of cardiovascular disease and the leading cause of death, which affects more than 40 million people worldwide (1–3). With the development of the global population growth and the acceleration of population aging, the absolute number of patients with heart failure has been increasing (4, 5). Meanwhile, the proportion of patients with HF with hypertension, atrial fibrillation, and diabetes increased significantly (6–8). Existing studies have found that diabetes could increase the risk of HF and lead to a poor prognosis for patients with HF, especially for those in intensive care unit (ICU) (4, 6, 9). Mechanistic hypotheses related to hyperglycemia, oxidative stress, or inflammation have been explored (10). Some researchers further found that the increased risk of events associated with diabetes was partially explained by structural and functional abnormalities of heart (11). However, the exact pathophysiological mechanisms have not been fully elucidated, and the specific treatment measures for patients with HF with diabetes still need to be further developed. Some clinically widely used severity score indicators, such as Simplified Acute Physiology Score II (SAPS-II) and Acute Physiology and Chronic Health Evaluation II (APACHE-II), were not specifically evolved for patients with HF (12, 13). Therefore, these indicators did not show any outstanding performance to predict mortality risk for these patients, especially for those high-risk patients with HF with diabetes.

In recent years, artificial intelligence (AI) has increasingly penetrated into the medical field (14). Through appropriate “learning”, computers can replace the human brain to deal with a large number of complex tasks. AI is capable of helping process image information, support diagnosis, recognize patterns of disease, and so on, so that clinicians could provide patients with better healthcare (14). Notably, unsupervised learning enables the discovery of latent structures or patient subgroups in specific cohorts, especially in ICU-related tasks (15). Some clinical decision support studies have demonstrated the ability of sophisticated machine learning models in solving certain ICU-related tasks and gained satisfying performance (16–19).

This is the first study that focused on predicting mortality risk for a specific group of high-risk populations, namely, patients with HF with diabetes in ICU. In Medical Information Mart for Intensive Care IV (MIMIC-IV) population, we used clustering algorithm to classify candidates into high-risk or low-risk groups, and then, nine machine learning models were employed to identify the major indicators for all-cause in-hospital mortality in these populations and two subgroups. Taking this as the cornerstone, a new composite indicator, ASL, was established and externally validated in the eICU cohort. Our study showed that ASL had a better performance in forecasting mortality risk in patients with HF with diabetes.

## Method

### Data Sources

This study used two high-quality large public databases. First is the MIMIC-IV database, which consisted of more than 53,000 patients in ICU between 2008 and 2019 at Beth Israel Deaconess Medical Center (20). The database contained the basic demographic information, vital signs, and biochemical indexes of each patient during ICU. Nurses recorded these data every other hour to ensure authenticity and reliability. Users were required to apply for and pass the test to obtain database permissions. Informed consent was not required for this database for all patient information was processed anonymously. Second is the eICU Collaborative Research Database, a multi-center emergency database, which included ICU records of more than 200,000 patients from 208 hospitals across the United States (21).

### Study Population and Study Design

**Figure 1:**
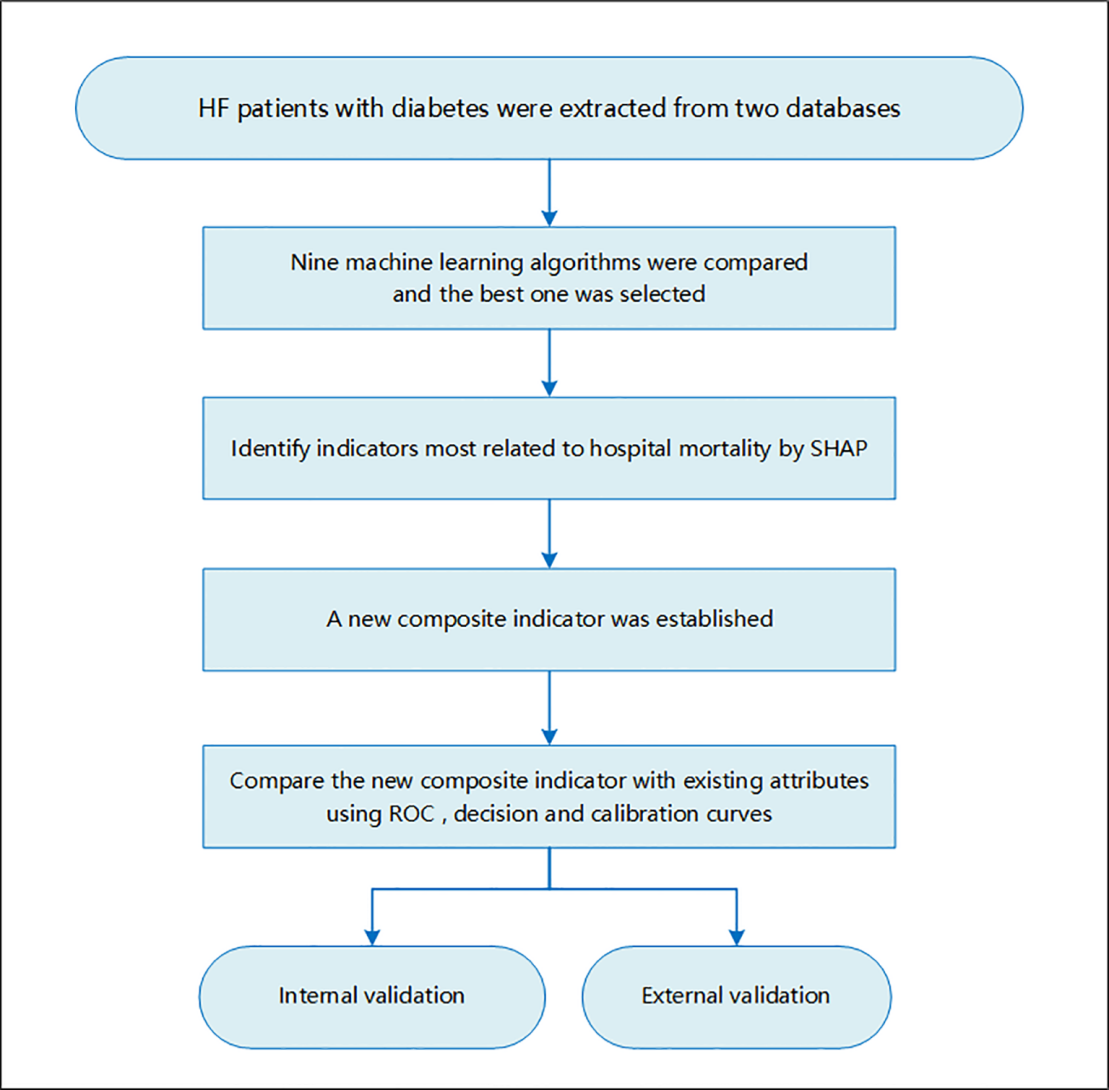
Flowchart of this study.

This study focused on critically ill patients with HF complicated with diabetes. The inclusion criteria of the study population were as followed: (1) 18 years old or older, (2) had experience in ICU, and (3) diagnosis of HF and diabetes. Those who had no ICU experience or stayed in ICU for less than 24 h were excluded. For patients with multiple admissions or ICU history, only the first ICU experience at the first admission was included. This study was a large multi-center cohort study, and the study flowchart is shown in **Figure 1**.

### Data Extraction and Preprocessing

SQL language and International Classification of Diseases and Ninth Revision (ICD-9) codes were used to extract data from the two databases. We enrolled patients admitted to ICU with a diagnosis of HF and diabetes on hospital admission. The patient’s basic demographic information, such as sex, age, and laboratory indicators like blood glucose, creatinine, and urea, were extracted one by one. Scores related to the severity of the disease, such as Sepsis-related Organ Failure Assessment (SOFA) Score, Systemic Inflammatory Response Syndrome (SIRS) criteria, Acute Physiology Score (APS) III, and some common comorbidities or drugs were also included in the final cohort. Data with missing values of more than 30% were deleted, and other vacant values were filled by multiple interpolation. This process was implemented in Stata (version 14.0). To find out all possible hidden connections, each continuous index was divided into three groups of Min, Max, and Mean. The Max or Min value referred to the maximum or minimum value of all the measured values of the attribute during this ICU stay. Mean represented the average of the maximum and minimum value. The primary outcome was all-cause in-hospital mortality.

### Machine Learning Model Comparisons and Identify Risk Indicators

Nine machine learning models were established and validated, including Logistic Regression, Support Vector Classifier (SVC), Decision Tree, Bagging, Gradient Boosting Machine (GBM), K-nearest neighbors (KNN), Random Forest, XGBoost, and LightGBM. A total of 80% of the study population was randomly selected as the training set, and the remaining 20% was used for internal validation. Each model was validated by five times cross-folding, and the average accuracy was obtained. We used areas under the receiver operating characteristic (ROC) curves (AUCs) to evaluate the performance of models as well as the precision and recall rate. The model with the best efficiency was further adjusted by hyper-parameters to optimize its performance. Then, a “perfect” model was established to define risk indicators most related to hospital mortality using SHAP in the three groups: patients with HF with diabetes, high-risk cohort, and low-risk cohort. All the steps were performed using Python.

Shapley Additive Explanations (SHAP) is a visual method to interpret the results of machine learning algorithm. We used SHAP to identify the top 20 indicators associated with in-hospital mortality based on machine learning models. This method assessed the importance of each feature using a game-theoretic approach (22). To obtain the importance of each feature at the overall level, the SHAP values of all features for all samples were drawn, and then, they were sorted in descending order according to the sum of the SHAP values. The color represents the importance of the feature (red represents high, and blue represents low), and each point represents a sample.

In addition, to further obtain the subgroups in the patient population, we used the R package called “ConsensusClusterPlus”. On the basis of this, we can further identify risk factors and test predictive effectiveness in more subdivided patient subgroups. This is an unsupervised clustering method based on the quantity of each index. To prevent the redundancy of work, we divided patients into high-risk groups and low-risk groups.

### Comparison Between the New Composite Indicator and Existing Attributes

After the new composite indicator was established by linear fitting using logistics regression, we introduced three analyses to compare the performance between the new indicator and existing attributes, including ROC curve, decision curve analysis (DCA), and calibration curve. The AUC curve only measures the diagnostic accuracy of the predictive model and fails to take into account the clinical utility of a specific model, whereas the advantage of DCA is that it integrates the preferences of patients or decision-makers into the analysis. In the calibration curve analysis, by drawing the fitting of the actual probability under different conditions and the probability predicted by the model, the evaluation of the prediction effect of the model on the actual results is judged.

### Statistical Analysis

Data were presented in the tables according to different distributions and types of variables. Categorical variables were presented as numbers (percentages) and tested by Chi-square (or Fisher’s exact) tests. Continuous variables were presented as mean ± standard deviation or median (25–75 percentiles) and were tested by student’s t-test or Wilcoxon rank sum tests. The composite indicator was generated using logistics regression, which was implemented in SPSS (version 23.0). To address the possibility of confounding differences and selection bias, propensity score matching (PSM) was performed using a 1:1 greedy nearest-neighbor algorithm within specified calliper widths. Locally weighted scatter plot smoothing (Lowess) could better deal with this problem by fitting a line in line with the overall trend, so as to better expose the hidden trend.

All statistical analyses in this study were performed using SPSS (version 23.0) or Stata (version 14.0). SHAP and machine learning algorithms were implemented using Python (version 3.9.7). Cluster analysis is implemented using R language (version 4.1.3) **(** **Supplementary Figure 1** **)**. Lowess and PSM were analyzed with Stata (version 14.0). A P-value lower than 0.05 was set for statistical significance in this study.

## Results

### Baseline Characteristics and Cluster Analysis

**Figure 2:**
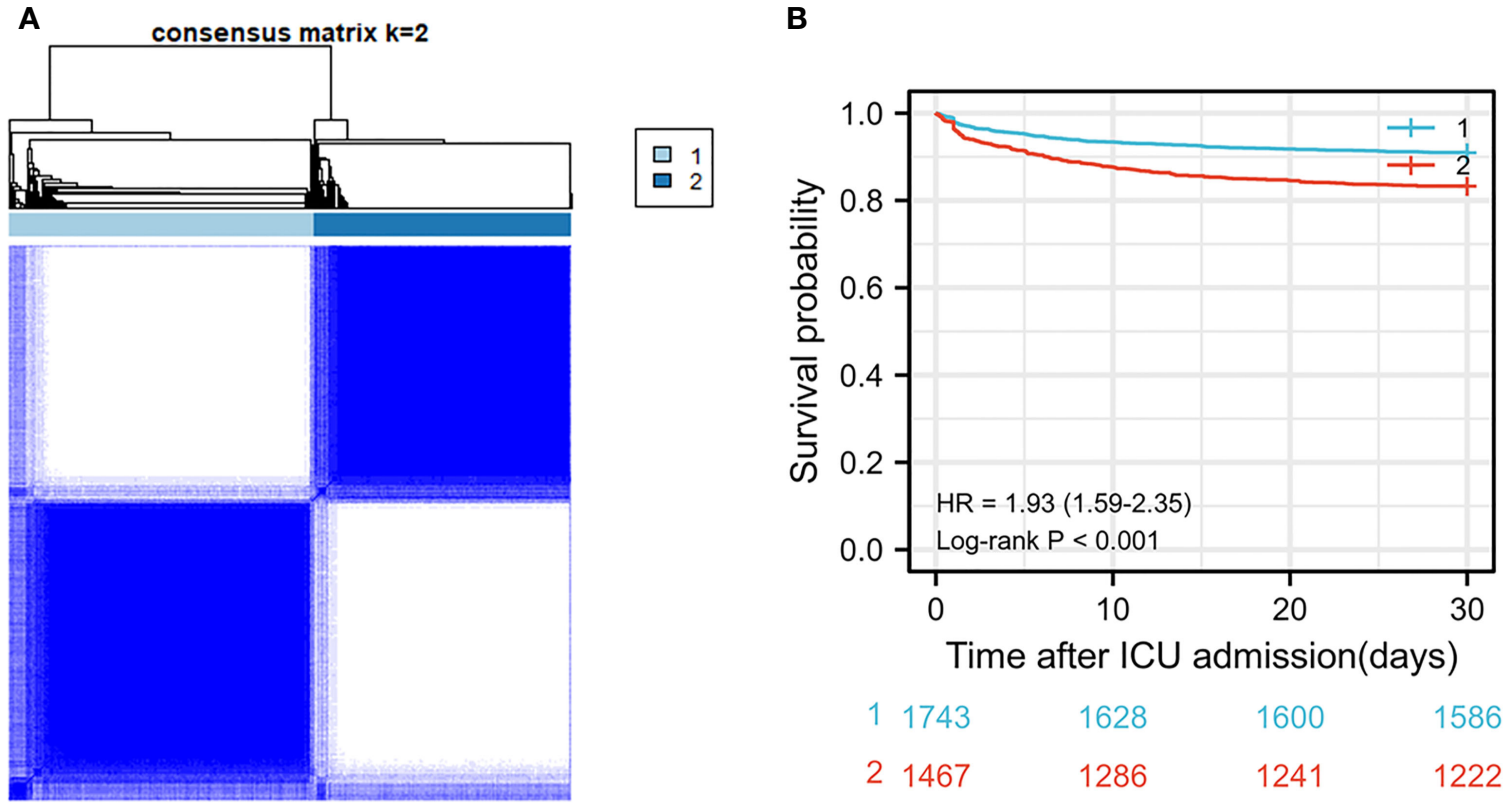
**(A)** Cluster diagram in MIMIC-IV population. **(B)** Survival curve between two clusters of patients in MIMIC-IV population.

After screening for inclusion and exclusion criteria, a total of 3,210 MIMIC-IV patients were included in the study cohort. As shown in **Supplementary Table 1**, 395 patients died during hospitalization, whereas 2,815 patients survived. After cluster analysis of study participants using R language, all patients were divided into two subgroups, namely, cluster 1 and cluster 2 (**Figure 2A**). Since entering ICU, the survival curves of the two clusters of patients were drawn and the log-rank test was less than 0.001 (**Figure 2B**). The risk of death in the cluster 2 patients was significantly higher than that in the cluster1 group with hazard ratio (HR) = 1.93 (1.59–2.35). Therefore, we defined cluster 1 as the low-risk group and the other one as the high-risk group. As the **Table 1** shown, the patients in high-risk group were older and consisted of more male patients. There was no significant difference in heart rate (HR) and Mean respiratory rate (RR) between these two groups. The difference of value between body temperature and SpO_2_ was mild, although there was a statistical difference between the two groups. Notably, overall, the systolic blood pressure, diastolic blood pressure, and mean blood pressure of the high-risk group were significantly lower than that of the low-risk group. In terms of biochemical indicators, the high-risk group had lower glucose, HbA1c, platelet, and bicarbonate, whereas blood urea nitrogen and creatinine were significantly higher than the other group, and urine output was lower, indicating that the high-risk group had a worse renal function. For some indexes, reflecting the degree of heart damage, the CK-MB, Troponin-T, and NT-Pro-BNP of the high-risk group were significantly higher than those in the low-risk group. For sodium, potassium, and other indicators, the two groups were very approximate in value.

**Table 1:** Baseline characteristics of the low-risk group and high-risk group after cluster analysis in MIMIC-IV cohort.

	Cluster 1 (Low-Risk Group, N = 1,743)	Cluster 2 (High-Risk Group, N = 1,467)	P-Value
Age (years)	72 (63–81)	75 (67–83)	<0.001
Males (n (%))	886 (50.8)	849 (57.9)	<0.001
Min HR (/min)	69 (60–79)	68 (60–78)	0.034
Max HR (/min)	98 (86–112)	96 (84–111)	0.189
Mean HR (/min)	82 (72–92)	81 (71–91)	0.075
Min RR (/min)	13 (11–15)	12 (10–15)	0.002
Max RR (/min)	28 (24–32)	27 (24–31)	0.028
Mean RR (/min)	19 (17–22)	19 (17–21)	0.002
Min Temperature (°C)	36.4 (36.1–36.7)	36.3 (35.9–36.5)	<0.001
Max Temperature (°C)	37.2 (36.9–37.6)	37.1 (36.8–37.4)	<0.001
Mean Temperature (°C)	36.8 (36.6–37.0)	36.7 (36.4–36.9)	<0.001
Min SpO_2_ (%)	92 (89–94)	92 (89–95)	0.758
Max SpO_2_ (%)	100 (99–100)	100 (100–100)	0.009
Mean SpO_2_ (%)	97 (95–98)	97 (96–99)	0.001
Min SBP (mmHg)	91 (82–102)	89 (80–100)	<0.001
Max SBP (mmHg)	148 (132–165)	143 (130–160)	<0.001
Mean SBP (mmHg)	118 (107–131)	114 (104–127)	<0.001
Min DBP (mmHg)	43 (37–50)	41 (35–48)	<0.001
Max DBP (mmHg)	86 (74–100)	81 (70–95)	<0.001
Mean DBP (mmHg)	60 (53–68)	57 (51–64)	<0.001
Min MBP (mmHg)	57 (51–64)	55 (49–62)	<0.001
Max MBP (mmHg)	100 (89–115)	97 (86–110)	<0.001
Mean MBP (mmHg)	75 (69–82)	73 (67–80)	<0.001
**Lab events**			
Min Glucose (mmol/L)	120 (93–152)	107 (82–140)	<0.001
Max Glucose (mmol/L)	216 (170–285)	200 (158–257)	<0.001
Mean Glucose (mmol/L)	164 (133–210)	148 (124–191)	<0.001
Min WBC (K/µl)	4.3 (3.0–5.9)	4.9 (3.1–6.5)	<0.001
Max WBC (K/µl)	18.5 (13.3–24.9)	17.7 (12.9–23.7)	0.068
Mean WBC (K/µl)	8.3 (6.6–10.7)	8.5 (6.7–11.0)	0.092
Min RBC (m/µl)	2.8 (2.4–3.1)	2.7 (2.4–3.0)	<0.001
Max RBC (m/µl)	4.6 (4.2–5.1)	4.3 (3.9–4.8)	<0.001
Mean RBC (m/µl)	3.5 (3.1–4.0)	3.4 (3.0–3.7)	<0.001
Min Platelet (K/µl)	133 (106–161)	126 (92–157)	0.001
Max Platelet (K/µl)	386 (294–493)	334 (251–440)	<0.001
Mean Platelet (K/µl)	231 (177–295)	203 (151–266)	<0.001
Min Hemoglobin (g/dl)	7.9 (6.8–8.9)	7.8 (6.9–8.7)	0.117
Max Hemoglobin (g/dl)	13.4 (12.6–14.4)	12.8 (11.7–13.7)	<0.001
Mean Hemoglobin (g/dl)	10.3 (9.1–11.3)	9.8 (9.0–10.8)	<0.001
Min anion gap (mEq/L)	9 (7–10)	10 (8–11)	<0.001
Max anion gap (mEq/L)	21 (19–25)	22 (19–26)	0.028
Mean anion gap (mEq/L)	15 (14–17)	16 (14–18)	<0.001
Min bicarbonate (mEq/L)	19 (16–22)	18 (15–21)	<0.001
Max bicarbonate (mEq/L)	34 (31–37)	32 (29–35)	<0.001
Mean bicarbonate (mEq/L)	26 (24–29)	25 (23–28)	<0.001
Min Sodium (mmol/L)	131 (127–134)	131 (127–135)	0.027
Max Sodium (mmol/L)	146 (143–148)	145 (142–148)	<0.001
Mean Sodium (mmol/L)	139 (137–141)	139 (136–141)	0.006
Min Potassium (mmol/L)	3.2 (3.0–3.5)	3.3 (3.0–3.6)	<0.001
Max Potassium (mmol/L)	5.9 (5.2–6.9)	5.7 (5.1–6.6)	<0.001
Mean Potassium (mmol/L)	4.3 (4.0–4.7)	4.4 (4.0–4.8)	<0.001
Min Chloride (mmol/L)	92 (87–96)	93 (88–97)	<0.001
Max Chloride (mmol/L)	110 (107–113)	110 (106–113)	0.258
Mean Chloride (mmol/L)	101 (99–104)	101 (98–104)	0.427
Min Calcium (mmol/L)	7.7 (7.2–8.2)	7.7 (7.2–8.1)	0.219
Max Calcium (mmol/L)	9.9 (9.5–10.3)	9.6 (9.2–10.2)	0.001
Mean Calcium (mmol/L)	9.0 (8.8–9.2)	8.9 (8.7–9.2)	<0.001
Min BUN (mg/dl)	12 (8–16)	16 (11–24)	<0.001
Max BUN (mg/dl)	59 (38–91)	73 (47–104)	<0.001
Mean BUN (mg/dl)	22 (17–32)	32 (22–46)	<0.001
Min Creatinine (mg/dl)	0.8 (0.6–1.0)	1.0 (0.8–1.4)	<0.001
Max Creatinine (mg/dl)	2.2 (1.5–3.6)	2.9 (1.8–5.2)	<0.001
Mean Creatinine (mg/dl)	1.1 (0.9–1.5)	1.5 (1.1–2.2)	<0.001
Min Lactate (mg/dl)	1.0 (0.8–1.2)	1.0 (0.8–1.4)	<0.001
Max Lactate (mg/dl)	3.2 (2.2–4.6)	2.9 (1.8–5.2)	0.153
Mean Lactate (mg/dl)	1.6 (1.2–2.1)	1.5 (1.1–2.1)	0.289
Min ALT (IU/L)	12 (8–17)	13 (9–21)	0.001
Max ALT (IU/L)	48 (28–123)	50 (25–191)	0.033
Mean ALT (IU/L)	22 (16–33)	23 (15–44)	<0.001
Min Bilirubin (mg/dl)	0.3 (0.2–0.4)	0.3 (0.2–0.5)	<0.001
Max Bilirubin (mg/dl)	0.8 (0.5–1.6)	1.0 (0.5–1.8)	0.002
Mean Bilirubin (mg/dl)	0.5 (0.3–0.7)	0.5 (0.3–0.9)	<0.001
Min Albumin (g/dl)	3.0 (2.6–3.5)	3.0 (2.5–3.4)	<0.001
Max Albumin (g/dl)	4.2 (3.8–4.5)	3.9 (3.5–4.3)	<0.001
Min Urine output (ml)	30 (12–62)	20 (5–40)	<0.001
Max Urine output (ml)	460 (300–680)	375 (240–500)	<0.001
Mean Urine output (ml)	200 (100–350)	120 (50–230)	<0.001
Min CK-MB (ng/ml)	2.0 (2.0–4.0)	3.0 (2.5–3.4)	<0.001
Max CK-MB (ng/ml)	7.0 (4.0–19.0)	9.0 (4.0–26.0)	0.010
Min Troponin-T (ng/ml)	0.02 (0.01–0.11)	0.06 (0.02–0.24)	<0.001
Max Troponin-T (ng/ml)	0.28 (0.07–1.19)	0.41 (0.11–1.66)	0.001
Min NT-Pro-BNP (pg/ml)	1,052 (381–2367)	6,817 (4,563–11,696)	<0.001
Max NT-Pro-BNP (pg/ml)	3,972 (1,797–8,848)	13,316 (8,786–23,503)	<0.001
Mean NT-Pro-BNP (pg/ml)	1,625 (607–3123)	9,068 (6,419–14,854)	<0.001
Min HbA1c (%)	6.3 (5.9–6.9)	6.3 (5.9–6.9)	0.187
Max HbA1c (%)	7.6 (6.8–9.3)	7.1 (6.5–8.1)	<0.001
Mean HbA1c (%)	6.9 (6.3–8.0)	6.7 (6.2–7.5)	<0.001
**Disease score**			
APS III	46 (37–60)	53 (43–68)	<0.001
SOFA	4 (2–7)	6 (4–8)	<0.001
SIRS	2 (2–3)	2 (2–3)	0.162
**Comorbidity**			
Hypertension (n (%))	1,044 (59.9)	864 (58.9)	0.588
Arrhythmia (n (%))	672 (38.6)	565 (38.5)	1.000
Cardiomyopathy (n (%))	405 (23.2)	350 (23.9)	0.707
Coronary disease (n (%))	1061 (60.9)	877 (59.8)	0.538
MI (n (%))	528 (30.3)	413 (28.2)	0.186
Peripheral vascular disease (n (%))	342 (19.6)	273 (18.6)	0.472
Cerebral disease (n (%))	280 (16.1)	236 (16.1)	1.000
Valvular disease (n (%))	404 (23.2)	301 (20.5)	0.072
COPD (n (%))	252 (14.5)	235 (16.0)	0.236
Respiratory failure (n (%))	430 (24.7)	369 (25.2)	0.774
Pulmonary heart diseases (n (%))	454 (26.0)	338 (23.0)	0.053
AKI (n (%))	1005 (57.7)	819 (55.8)	0.300
CKD (n (%))	922 (52.9)	752 (51.3)	0.357
Hyperlipidemia (n (%))	1163 (66.7)	948 (64.6)	0.218
Hypothyroidism (n (%))	330 (18.9)	280 (19.1)	0.928
Hemopathy (n (%))	467 (26.8)	385 (26.2)	0.748
**Drug use**			
Insulin (n (%))	549 (31.5)	432 (29.4)	0.218
Loop diuretic (n (%))	1,354 (77.7)	1,154 (78.7)	0.520
β-blocker (n (%))	1,205 (69.1)	1,006 (68.6)	0.760
Digoxin (n (%))	138 (7.9)	132 (9.0)	0.278
Albumin (n (%))	263 (15.1)	211 (14.4)	0.583
Dobutamine (n (%))	66 (3.8)	49 (3.3)	0.507
ACEI/ARB (n (%))	664 (38.1)	516 (35.2)	0.091
Epinephrine (n (%))	128 (7.3)	86 (5.9)	0.102
Norepinephrine (n (%))	409 (23.5)	323 (22.0)	0.332
CCB (n (%))	291 (16.7)	241 (16.4)	0.849

HR, heart rate; RR, respiratory rate; SBP, systolic blood pressure; DBP, diastolic blood pressure; MBP, mean blood pressure; WBC, white blood cell; RBC, red blood cell; BUN, blood urea nitrogen; ALT, alanine aminotransferase; CK-MB, creatine kinase-MB; NT-Pro-BNP, N-terminal pro-B-type natriuretic peptide; APS, Acute Physiology Score; SOFA, Sepsis-related Organ Failure Assessment; SIRS, Systemic Inflammatory Response Syndrome; MI, myocardial infarction; COPD, chronic obstructive pulmonary disease; AKI, acute kidney injury; CKD, chronic kidney disease; ACEI, angiotensin-converting enzyme inhibitors; ARB, angiotensin receptor blockers; CCB, Calcium channel blocker.

### Development and Comparison of Machine Learning Models

**Figure 3:**
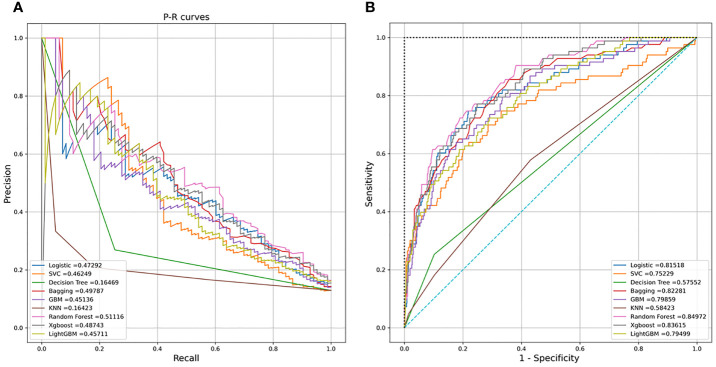
**(A)** Receiver operating characteristic (ROC) curves of the nine models. **(B)** Precision-Recall (P-R) curves of the nine models.

**Figure 4:**
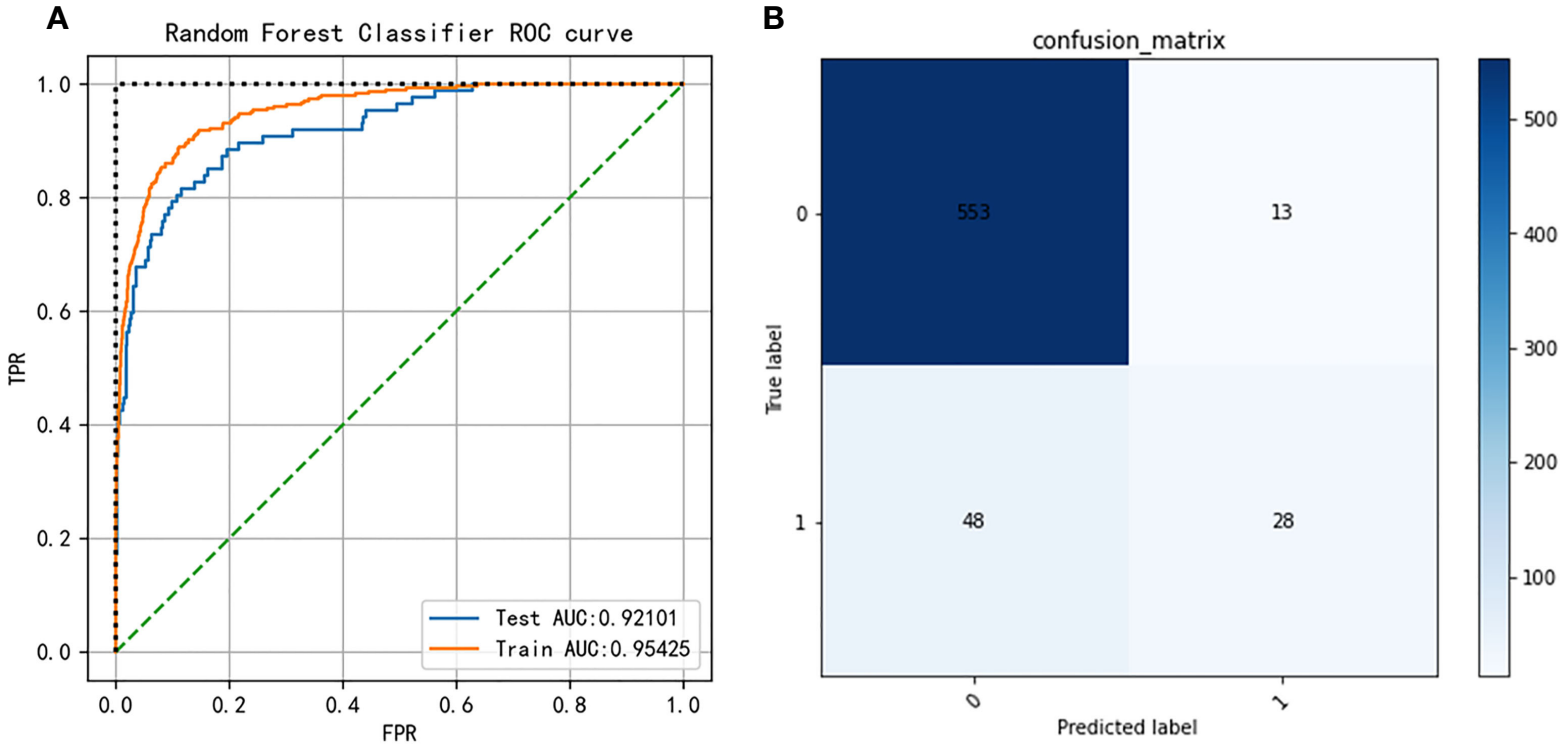
**(A)** Receiver operating characteristic (ROC) curves of the Random Forest model after hyper-parameter optimization. **(B)** Confusion matrix of the Random Forest model after hyper-parameter optimization.

Nine machine learning models were employed in this study, including Logistic Regression, SVC, Decision Tree, Bagging, GBM, KNN, Random Forest, XGBoost, and LightGBM. These were all commonly used models for solving binary classification problems. Each model was verified by five cross-fold validation, and their AUC and Precision-Recall (P-R) curves were drawn in **Figure 3**. Among them, the random forest algorithm had the finest discrimination ability with precision = 0.511 and AUC = 0.850 (**Table 2**), so we chose it to establish the final model. After hyper-parameter optimization using grid and random hyper-parameter search, the final random forest model reached AUC = 0.92, and the confusion matrix was displayed (**Figure 4**). All demographic information, vital signs, laboratory indicators, complications, and drug medications were included in the final analysis. Whereafter, we respectively analyzed the related factors of the overall population, high-risk group, and low-risk group using the final RM algorithm.

**Table 2:** Comparisons of nine different machine learning models.

Model	Precision	AUC
Logistics regression	0.473	0.815
SVC	0.462	0.752
Decision tree	0.165	0.576
Bagging	0.498	0.823
GBM	0.451	0.799
KNN	0.164	0.584
Random Forest	0.511	0.850
XGBoost	0.487	0.836
LightGBM	0.457	0.795

### Major Indicators Defined by SHAP

**Figure 5:**
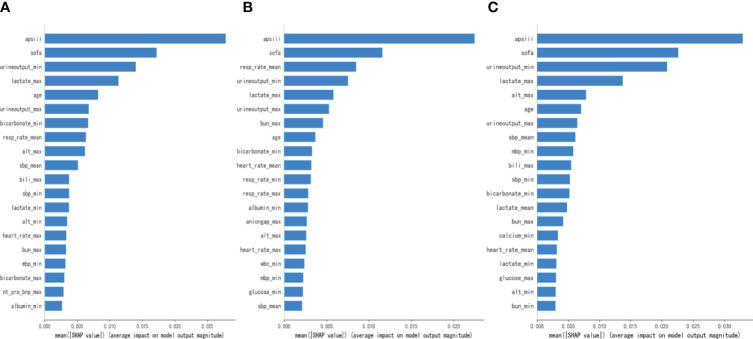
Bar charts that rank the importance of 20 indicators identified by Shapley Additive Explanations (SHAP) values. **(A)** The overall MIMIC-IV population. **(B)** Low-risk group. **(C)** High-risk group.

**Figure 6:**
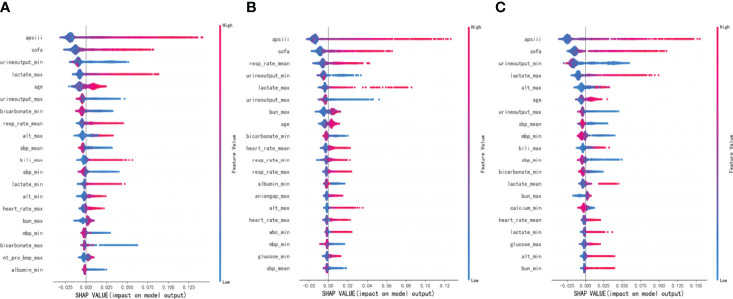
Distribution of the impact each feature had on the full model output using Shapley Additive Explanations (SHAP) values. **(A)** The overall MIMIC-IV population. **(B)** Low-risk group. **(C)** High-risk group.

To make the output of the model more visual, we introduced SHAP to identify the factors that have the greatest correlation with hospital mortality. As shown in **Figure 5**, for the entire population, a total of 20 factors were identified. Among them, the top five indicators were APS III, SOFA, Min urine output, Max lactate, and age. After that, we analyzed the low-risk group and high-risk group, respectively. Among the top five factors in the low-risk group, the only factor that was different from the overall population was Mean RR. Interestingly, in the high-risk group, Mean RR was not significantly associated with hospital mortality but was replaced by Max ALT. From the SHAP plot (**Figure 6**), a rough but imprecise trend could be observed. Among the three groups, patients with higher APS III, SOFA, Max lactate, and lower Min urine output had a greater risk of death. In the low-risk group, the higher Mean RR corresponded to the higher risk of death, whereas in the high-risk group, it was replaced by Max ALT.

### Establishment of a New Composite Indicator and Internal Validation

**Figure 7:**
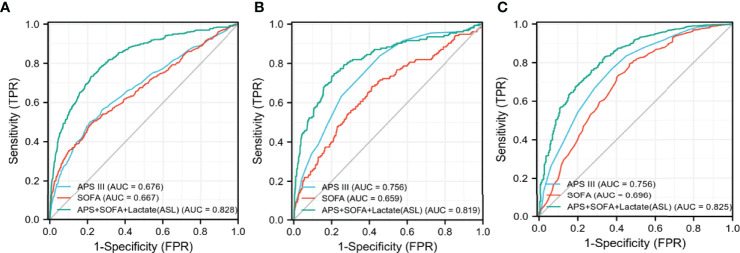
Receiver operating characteristic (ROC) curves of three different indicators in MIMIC-IV cohort. **(A)** The overall MIMIC-IV population. **(B)** Low-risk group. **(C)** High-risk group.

As shown in **Figures 5**, **6**, APS III, SOFA, and Max lactate were common indicators associated with in-hospital mortality in patients with HF with diabetes and two subclusters. On the basis of the three indicators mentioned above, logistics regression was employed to establish a novel composite indicator, which was named ASL. We validated this new indicator in MIMIC-IV cohort and found that, compared with APS III and SOFA, ASL had a more significant enhancement in predicting mortality risk in patients with HF with diabetes with AUC = 0.828 (**Figure 7**), independent of high-risk or low-risk group.

### External Validation in the eICU Cohort

To further confirm the predictive ability of ASL, we extracted patients with HF with diabetes from a multi-center database for external validation, namely, the eICU database.

**Figure 8:**
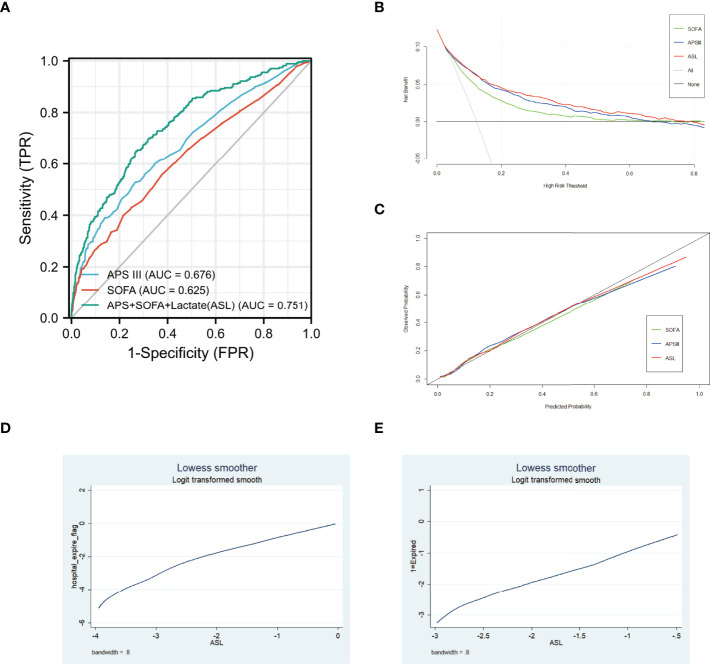
**(A)** Receiver operating characteristic (ROC) curves of three different indicators in eICU cohort. **(B)** DCA curves of three different indicators in eICU cohort. **(C)** Calibration curves of three different indicators in eICU cohort. **(D)** Association between ASL and hospital mortality in MIMIC-IV cohort using Lowess. **(E)** Association between ASL and hospital mortality in eICU cohort using Lowess.

A total of 3,862 patients were included in the eICU cohort. As shown in **Table 3**, non-survivors were older and had higher lactate, SOFA, and APS III. Compared with APS III and SOFA, ROC curve showed that ASL had a favorable performance in this external validation cohort, and the DCA curve, along with calibration curve, indicated that this indicator also had respectable clinical value (**Figures 8A–C** **)**. Taken together, this novel predictive indicator had acceptable sensitivity and specificity either in the derivation and validating cohort with a promising clinical value.

**Table 3:** Baseline characteristics of the survivors and non-survivors in eICU cohort.

	All patients(N = 3,862)	Survivors(N = 3,315)	Non-survivors(N = 501)	P-value
Age (years)	70 (61–77)	70 (60–77)	71 (65–79)	<0.001
Males (n (%))	2090 (54.12)	1,790 (85.65)	300 (14.35)	0.712
Min Lactate (mmol/L)	1.62 (1.00–2.29)	1.60 (1.00–2.20)	1.70 (1.10–2.54)	<0.001
Max Lactate (mmol/L)	2.84 (1.50–4.16)	2.80 (1.50–4.00)	3.20 (1.80–5.30)	<0.001
Mean Lactate (mmol/L)	2.12 (1.30–3.00)	2.10 (1.25–2.90)	2.37 (1.50–3.65)	<0.001
SOFA	4 (2–6)	3 (2–6)	6 (3–9)	<0.001
APS III	46 (34–61)	44 (33–58)	60 (44–82)	<0.001

APS, Acute Physiology Score; SOFA, Sepsis-related Organ Failure Assessment.

### Association Between ASL and Hospital Mortality Using Lowess

To further discover the exact relationship between major indicators and hospital mortality, we used the Lowess curve to analyze the overall population. For the general population, the relationship between APS III, SOFA, Max Lactate, and hospital mortality was approximately linear as a whole (**Supplementary Figure 2**). After using logistics regression to generate ASL, it is not surprising that there was also a linear positive correlation between ASL and mortality in MIMIC-IV cohort and eICU cohort (**Figures 8D, E** **)**. Then, we divided the patients into low-risk, middle-risk, and high-risk groups according to ASL and found that there were significant differences of mortality among the three groups both in MIMIC-IV cohort and eICU cohort, which showed that this indicator had great risk stratification ability (**Table 4**). To further confirm the relationship between drug medication and prognosis, patients were divided into medication group and non-medication group according to 1:1 matching. All 20 related factors were corrected by PSM. As displayed in **Supplementary Tables 2**, **3**, the use of diuretics and insulin had no significant effect on hospital mortality.

**Table 4:** Risk stratification using ASL in MIMIC-IV and eICU cohort.

Risk Stratification	Total Patients	Non-Survivors	Hospital Mortality
**MIMIC-IV cohort**			
Low risk	1,748	61	3.5%
Middle risk	920	142	15.4%
High risk	218	83	38.1%
**eICU cohort**			
Low risk	1,644	123	7.5%
Middle risk	1,349	181	13.4%
High risk	486	130	26.7%

## Discussion

In this study, we developed a novel composite indicator for predicting hospital mortality for patients with HF with diabetes. The AUCs of ROC curves demonstrated that, compared with SOFA and APS III, ASL had greater risk discrimination ability in these patients, independent of high-risk or low-risk groups. DCA and calibration curve further ensured the effectiveness and security of this indicator. Compared with existing attributes, this study proved that this novel composite indicator had a distinctive mortality risk prediction ability for this specific population and provided potential guiding values for clinical healthcare in ICU.

With the development of AI, machine learning has been more and more applied in the field of cardiovascular medicine, especially for patients with HF. Current HF diagnosis and management rely on physical examination, both laboratory and imaging data of patients (23). The advantage of machine learning is that it can learn from vast amounts of existing data and output the most valuable results. For example, machine learning has been applied to the diagnosis of HF, the prediction of mortality, and readmission rate and achieved good performance (24–30). Previous studies have also confirmed that the random forest model had outstanding ability to identify risk factors in patients with HF, and the left ventricular ejection fraction was successfully identified as the most relevant feature in predicting the mortality risk of patients (31). In our study, the random forest model stood out among the nine algorithms, which proved that it had the best prediction ability for this specific population. Our study is the first to apply machine learning algorithms to patients with HF with diabetes in the environment of ICU. Even with the development of medical treatment, the mortality rate in ICU remained at a high level with 11.3% in 1996 and 12.0% in 2010 (32). Therefore, predicting the mortality risk of critically ill patients could provide useful guidance for clinical healthcare.

Cardiovascular disease caused 18 million deaths worldwide each year, and the coexistence of diabetes made cardiovascular mortality risk higher (33, 34). Meanwhile, diabetes, especially type 2 diabetes, affected more than 400 million people worldwide (35). Its pathophysiological mechanism has been widely studied, and it has been proved that it is closely related to microvascular and macrovascular complications, especially for the development of HF (36, 37). Therefore, the number of patients with these two common diseases had been already quite widespread and the risk of death might be greatly increased in patients with HF with diabetes. However, there is no risk assessment tool for this type of patient, especially for critical ill patients. Currently, there are many ICU scoring systems, whereas the predictive effect of these scoring systems on the mortality risk varies among different populations, including Acute Physiology and Chronic Health Evaluation (APACHE) (II, III, and IV), SIRS criteria, and SOFA score (38, 39). In terms of their purposes of creation and previous related studies, although they can estimate patients’ conditions quickly within 10 min so that doctors can acquire clinical dynamics of disease changes and give feedback strategies, due to the heterogeneity of the patient population, the performance of the existing scoring system in common use had inevitably volatility (40–43), for example, the SIRS may lack sufficient sensitivity and specificity to identify and risk-stratify patients in some cases. To ensure the monitoring ability of common use scoring systems for HF with diabetes patients, our study selected the APS III, SOFA, and SIRS, which were commonly used in ICU in America. We found that APS III and SOFA performed best in both high-risk and low-risk groups, whereas SIRS performed poorly. APS III was designed to predict the in-hospital mortality of ICU patients; focused on the lowest score of several vital signs, laboratory examinations, and nervous system in the first 24 h; and has been widely used to predict the clinical outcome of mixed critically ill patients now (44, 45). SOFA could describe the dysfunction or failure of one or more organs and evaluate the degree from mild dysfunction to severe failure, from repeated measures of the occurrence and progression of dysfunction in one or all organs. The items in SOFA are continuous variables that are objective, accessible, and reliable to avoid confusion and bias from the source of patients, entities, and demographics (46). Therefore, these two scoring systems complement each other in ASL and fully demonstrate their ability for real-time assessment and long-term dynamic monitoring in the time dimension. Moreover, it not only includes intuitive results such as vital signs and laboratory tests but also objectively collects the changes of various tissues and organs, so as to pay more attention to the overall changes in the spatial dimension (47). Although both urine output and lactate were identified to be highly correlated with hospital mortality based on random forest model, only lactate was selected in the composite indicator for urine output was already included in the APS III score, whereas lactate was not. Lactate, an end-stage product of anaerobic cell metabolism, always occurs during hypoxic conditions and has been reported to be associated with multiple organ dysfunction, poor prognosis, and higher in-hospital mortality. The metabolism of glucose in sensitive tissues is severely altered in diabetes patients or patients with HF who are in a state of oxygen imbalance and depletion, including defective glycogen synthesis and impaired glucose oxidative metabolism, following multiple tissues and organs that act negatively in processing the elevated lactate concentration so that the production of lactic acid increased with the increase of non-oxidized glycolysis in blood (48, 49). Because of the exquisite recognition ability of the machine learning model, the final composite indicator performed better than the existing scoring system in predicting mortality risk in patients with HF with diabetes.

At present, there is no in-depth study on the specific treatment measures for this kind of patient. As displayed in **Supplementary Tables 2**, **3**, we found that diuretics and insulin did not significantly improve the prognosis of these patients after PSM, which indicated that these patients might have unelucidated pathophysiological mechanisms and required more specific treatment. Sodium-glucose co-transporter 2 inhibitors (SGLT2is), which are initially introduced as oral anti-diabetic drugs to reduce blood glucose by inhibition of sodium-glucose cotransporters in kidney, are now known to reduce the combined risk of cardiovascular death in patients with HF with or without diabetes (50, 51). By combining with ASL indicator, we could identify high-risk patients and improve their clinical treatment strategies, such as replacing or adding SGLT2 drugs. The effect of those promising drugs on critical ill patients remained to be further studied in the future.

There were several limitations to this study. First, this was a retrospective study, although we used two databases of multiple centers for internal and external validation respectively, more extensive research studies were still required in the future. Second, there were multiple subtypes of HF and diabetes, which were not subdivided in this study. Nevertheless, this study was the first to focus on patients with HF with diabetes in a critical care environment and was expected to help improve the prognosis of these patients in the future.

## Conclusion

In this study, we developed a novel composite indicator for predicting hospital mortality for patients with HF with diabetes admitted to ICU, which was validated in internal and external cohorts. Compared with existing attributes such as APS III and SOFA, the new indicator had better discrimination ability and clinical value, which had potential value in reducing the mortality risk of these patients.

## Data Availability Statement

Subject to the databases’ license, the raw data supporting the conclusions of this article will be made available by the authors, without undue reservation.

## Ethics Statement

Ethical review and approval was not required for the study on human participants in accordance with the local legislation and institutional requirements. Written informed consent for participation was not required for this study in accordance with the national legislation and the institutional requirements.

## Author Contributions

BY and YZ conceived the theme and wrote the manuscript. CS and XL improved the manuscript. All authors contributed to the article and approved the submitted version.

## Funding

CS was supported by the National Natural Science Foundation of China (81871105) and the Shanghai Shenkang Hospital Development Center (SHDC2020CR1042B).

## Conflict of Interest

The authors declare that the research was conducted in the absence of any commercial or financial relationships that could be construed as a potential conflict of interest.

## Publisher’s Note

All claims expressed in this article are solely those of the authors and do not necessarily represent those of their affiliated organizations, or those of the publisher, the editors and the reviewers. Any product that may be evaluated in this article, or claim that may be made by its manufacturer, is not guaranteed or endorsed by the publisher.

## Supplementary Material

Supplementary Figure 1 Consensus index and Delta area of cluster analysis.

Supplementary Figure 2 Association between SOFA, APS III, Max Lactate and hospital mortality in MIMIC-IV cohort using Lowess.
