# Supplementary figures and images for: A Novel Composite Indicator of Predicting Mortality Risk for Heart Failure Patients With Diabetes Admitted to Intensive Care Unit Based on Machine Learning
Source: Front Endocrinol (Lausanne). 2022 Jun 29;13:917838. doi: 10.3389/fendo.2022.917838 (PMC9277005; doi:10.3389/fendo.2022.917838)

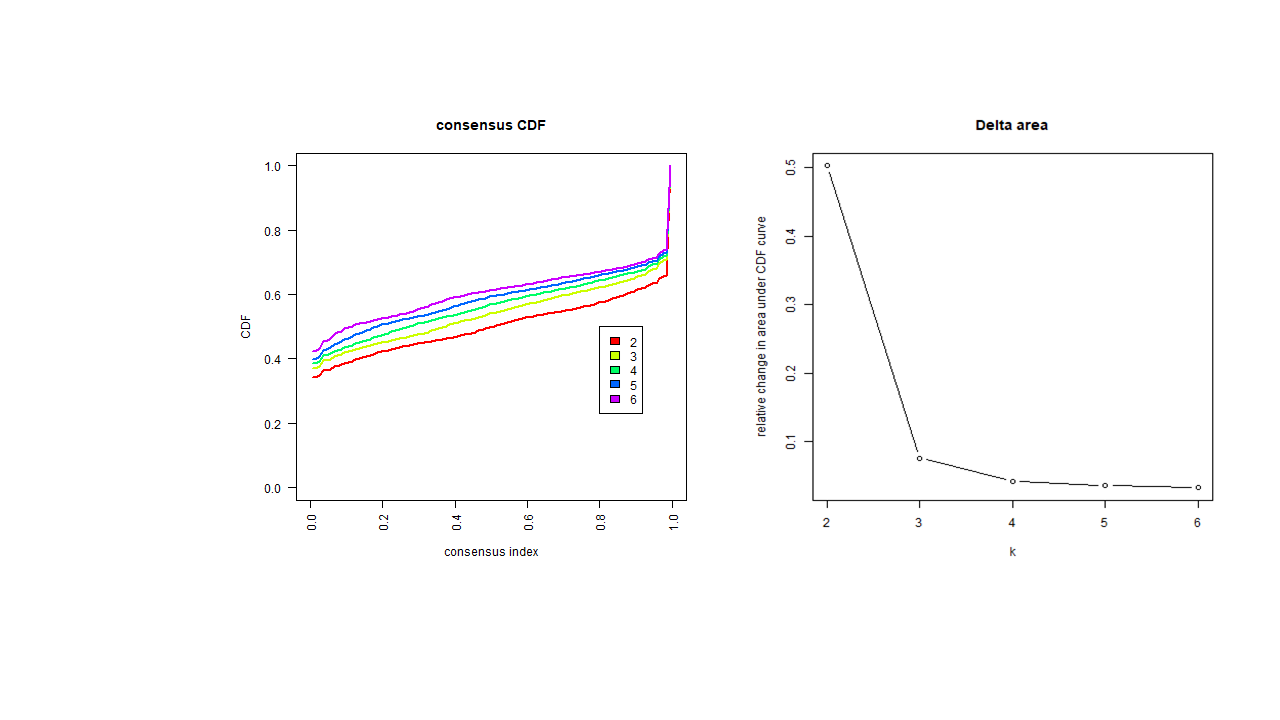

Supplement: Supplementary Figure 1 — Consensus index and Delta area of cluster analysis. [file Image_1.tif]

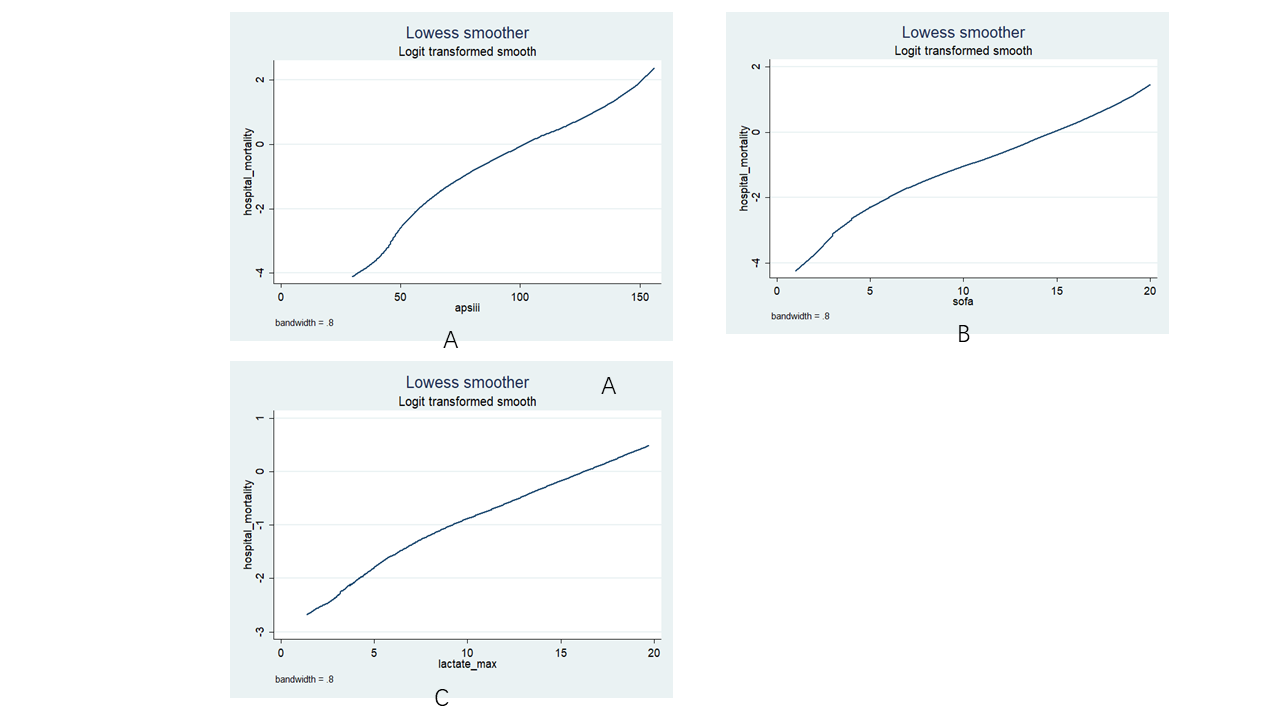

Supplement: Supplementary Figure 2 — Association between SOFA, APS III, Max Lactate and hospital mortality in MIMIC-IV cohort using Lowess. [file Image_2.tif]
