# Supplementary material for: A Novel Composite Indicator of Predicting Mortality Risk for Heart Failure Patients With Diabetes Admitted to Intensive Care Unit Based on Machine Learning
Source: Front Endocrinol (Lausanne). 2022 Jun 29;13:917838. doi: 10.3389/fendo.2022.917838 (PMC9277005; doi:10.3389/fendo.2022.917838)
Supplement: Supplementary file 3 [file Table_1.docx]

**Supplemental Table 1 Baseline characteristics between survivors and non-survivors in Mimic-IV cohort**

|  | All patients  （n=3210） | Survivors  (N=2815) | Non-survivors  (N=395) | P-Value |
| --- | --- | --- | --- | --- |
| Age (years） | 74(65-82) | 73(64-82) | 77(70-85) | ＜0.001 |
| Males(n(%)) | 1735(54.0) | 1517(53.9) | 218(55.1) | 0.666 |
| Min HR (/min) | 69(60-79) | 68(60-78) | 70(60-84) | 0.001 |
| Max HR(/min) | 97(85-111) | 96(85-110) | 104(90-120) | ＜0.001 |
| Mean HR(/min) | 81(72-92) | 81(71-91) | 86(73-98) | ＜0.001 |
| Min RR(/min) | 13(10-15) | 13(10-15) | 13(11-16) | 0.008 |
| Max RR(/min) | 27(24-31) | 27(24-31) | 29(25-33) | ＜0.001 |
| Mean RR(/min) | 19(17-22) | 19(17-21) | 20(18-24) | ＜0.001 |
| Min Temperature(℃) | 36.4(36-36.6) | 36.4(36.0-36.6) | 36.3(35.8-36.6) | 0.001 |
| Max Temperature(℃) | 37.1(36.9-37.5) | 37.1(36.9-37.5) | 37.1(36.7-37.5) | 0.109 |
| Mean Temperature(℃) | 36.7(36.5-37.0) | 36.7(36.5-37.0) | 36.7(36.4-36.9) | 0.023 |
| Min SpO2(%) | 92(89-94) | 92(90-94) | 91(87-94) | ＜0.001 |
| Max SpO2(%) | 100(99-100) | 100(99-100) | 100(100-100) | 0.315 |
| Mean SpO2(%) | 97(95-98) | 97(96-98) | 97(95-98) | 0.874 |
| Min SBP(mmHg) | 90(81-101) | 91(82-102) | 85(74-94) | ＜0.001 |
| Max SBP(mmHg) | 146(131-163) | 146(132-164) | 142(124-157) | ＜0.001 |
| Mean SBP(mmHg) | 116(106-130) | 117(107-130) | 109(101-123) | ＜0.001 |
| Min DBP(mmHg) | 42(36-49) | 43(36-49) | 40(32-47) | ＜0.001 |
| Max DBP(mmHg) | 84(72-98) | 84(72-98) | 82(72-97) | ＜0.001 |
| Mean DBP(mmHg) | 59(52-66) | 59(52-66) | 58(51-64) | 0.001 |
| Min MBP(mmHg) | 56(50-64) | 57(50-64) | 53(44-60) | ＜0.001 |
| Max MBP(mmHg) | 99(88-113) | 99(88-113) | 97(86-113) | 0.222 |
| Mean MBP(mmHg) | 74(68-82) | 75(68-82) | 72(66-78) | ＜0.001 |
| **Lab events** |  |  |  |  |
| Min Glucose(mmol/L) | 114(88-146) | 113(88-145) | 119(91-157) | 0.011 |
| Max Glucose(mmol/L) | 208(164-274) | 207(163-271) | 212(168-294) | 0.04 |
| Mean Glucose(mmol/L) | 157(128-202) | 154(127-200) | 171(134-216) | ＜0.001 |
| Min WBC (K/uL) | 4.6(3.0-6.2) | 4.6(3.0-6.1) | 4.7(3.0-6.6) | 0.152 |
| Max WBC (K/uL) | 18.3(13.1-24.3) | 17.9(12.9-24.1) | 19.8(14.9-25.8) | ＜0.001 |
| Mean WBC (K/uL) | 8.4(6.7-10.8) | 8.4(6.7-10.7) | 8.7(6.8-12.2) | 0.007 |
| Min RBC(m/uL) | 2.7(2.4-3.1) | 2.7(2.4-3.1) | 2.7(2.3-3.0) | 0.466 |
| Max RBC(m/uL) | 4.5(4.0-5.0) | 4.5(4.0-5.0) | 4.5(4.0-5.0) | 0.321 |
| Mean RBC(m/uL) | 3.4(3.1-3.9) | 3.4(3.1-3.9) | 3.5(3.0-3.9) | 0.999 |
| Min Platelet(K/uL) | 130(100-159) | 131(101-160) | 127(87-158) | 0.020 |
| Max Platelet(K/uL) | 362(273-471) | 364(274-475) | 354(258-454) | 0.008 |
| Mean Platelet(K/uL) | 218(164-283) | 218(166-283) | 210(154-278) | 0.112 |
| Min Hemoglobin(g/dL) | 7.9(6.9-8.0) | 7.9(6.9-8.8) | 7.9(6.9-8.7) | 0.819 |
| Max Hemoglobin(g/dL) | 13.2(12.2-14.1) | 13.2(12.2-14.2) | 13.1(12.1-13.9) | 0.076 |
| Mean Hemoglobin(g/dL) | 10.0(9.0-11.0) | 10.0(9.0-11.1) | 10.1(9.0-11.2) | 0.865 |
| Min aniongap | 9(8-11) | 9.0(8.0-11.0) | 10.0(8.0-12.0) | ＜0.001 |
| Max aniongap | 22(19-25) | 21.0(19.0-25.0) | 24.0(20.0-28.0) | ＜0.001 |
| Mean aniongap | 15(14-17) | 15.1(14.0-17.0) | 15.7(14.0-18.0) | 0.082 |
| Min bicarbonate | 19(15-21) | 19.0(16.0-22.0) | 16(13-20) | ＜0.001 |
| Max bicarbonate | 33(30-36) | 33.0(30.0-37.0) | 32(29-36) | ＜0.001 |
| Mean bicarbonate | 26(23-28) | 26.0(23.8-28.0) | 26(22-28) | 0.067 |
| Min Sodium(mmol/L) | 131(127-134) | 131(127-134) | 131(127-134) | 0.162 |
| Max Sodium(mmol/L) | 145(143-148) | 145(143-148) | 146(143-149) | 0.032 |
| Mean Sodium(mmol/L) | 139(136-141) | 139(136-141) | 138(136-141) | 0.037 |
| Min Potassium(mmol/L) | 3.3(3-3.5) | 3.2(3.0-3.5) | 3.3(3.0-3.6) | 0.129 |
| Max Potassium(mmol/L) | 5.8(5.2-6.7) | 5.8(5.1-6.8) | 5.9(5.3-6.7) | 0.114 |
| Mean Potassium(mmol/L) | 4.3(4.0-4.7) | 4.3(4.0-4.7) | 4.4(4.0-4.8) | 0.070 |
| Min Chloride(mmol/L) | 92(88-96) | 92(88-96) | 92(89-97) | 0.163 |
| Max Chloride(mmol/L) | 110(106-113) | 110(106-113) | 110(106-114) | 0.584 |
| Mean Chloride(mmol/L) | 101(98-104) | 101(99-104) | 101(98-104) | 0.311 |
| Min Calcium(mmol/L) | 7.7(7.2-8.1) | 7.7(7.2-8.2) | 7.5(6.9-7.9) | ＜0.001 |
| Max Calcium(mmol/L) | 9.8(9.3-10.2) | 9.8(9.3-10.2) | 9.8(9.2-10.3) | 0.305 |
| Mean Calcium(mmol/L) | 9.0(8.7-9.2) | 9.0(8.8-9.2) | 8.9(8.6-9.1) | ＜0.001 |
| Min BUN (mg/dL) | 13(9-20) | 13(9-19) | 16(11-24) | ＜0.001 |
| Max BUN (mg/dL) | 65(41-97) | 63(40-96) | 77(55-106) | ＜0.001 |
| Mean BUN (mg/dL) | 26(19-39) | 26(18-38) | 29(21-42) | ＜0.001 |
| Min Creatinine(mg/dL) | 0.9(0.6-1.2) | 0.8(0.6-1.2) | 1.0(0.7-1.3) | ＜0.001 |
| Max Creatinine(mg/dL) | 2.5(1.6-4.3) | 2.4(1.6-4.2) | 3.1(2.1-4.8) | ＜0.001 |
| Mean Creatinine(mg/dL) | 1.2(0.9-1.8) | 1.2(0.9-1.7) | 1.4(1.0-2.0) | ＜0.001 |
| Min Lactate(mg/dL) | 1.0(0.8-1.2) | 1.0(0.8-1.2) | 1.1(0.8-1.4) | ＜0.001 |
| Max Lactate(mg/dL) | 3.2(2.2-4.7) | 3.1(2.1-4.3) | 4.7(2.9-8.6) | ＜0.001 |
| Mean Lactate(mg/dL) | 1.5(1.1-2.1) | 1.5(1.1-2.1) | 1.7(1.3-2.6) | ＜0.001 |
| Min ALT | 12(8-19) | 12(8-18) | 14(10-20.8) | 0.005 |
| Max ALT | 49(26-150) | 46(26-131) | 86(37-264) | ＜0.001 |
| Mean ALT | 22(16-38) | 22(16-36) | 24(16-49) | ＜0.001 |
| Min Bilirubin | 0.3(0.2-0.5) | 0.3(0.2-0.5) | 0.4(0.2-0.6) | ＜0.001 |
| Max Bilirubin | 0.9(0.5-1.7) | 0.9(0.5-1.6) | 1.3(0.7-2.5) | ＜0.001 |
| Mean Bilirubin | 0.5(0.3-0.8) | 0.5(0.3-0.8) | 0.5(0.3-0.9) | 0.004 |
| Min Albumin | 3.0(2.5-3.4) | 3.0(2.6-3.4) | 2.8(2.3-3.3) | ＜0.001 |
| Max Albumin | 4.0(3.7-4.4) | 4.1(3.7-4.4) | 3.9(3.4-4.3) | ＜0.001 |
| Min Urine output | 25(10-50) | 28(10-50) | 5(0-21) | ＜0.001 |
| Max Urine output | 400(290-600) | 425(300-600) | 320(150-500) | ＜0.001 |
| Mean Urine output | 160(75-300) | 175(80-300) | 100(38-200) | ＜0.001 |
| Min CK | 2(2-4) | 2(2-4) | 3(2-5) | 0.025 |
| Max CK | 8(4-22) | 8(4-21) | 10(4-32) | ＜0.001 |
| Min Troponin-T | 0.04(0.02-0.16) | 0.04(0.02-0.16) | 0.04(0.02-0.18) | 0.401 |
| Max Troponin-T | 0.33(0.09-1.41) | 0.32(0.08-1.35) | 0.44(0.09-1.57) | 0.071 |
| Min Pro-BNP | 2987(882-6355) | 2765(817-6051) | 4803(1548-9673) | ＜0.001 |
| Max Pro-BNP | 8237(3451-16274) | 7943(3293-15473) | 11323(5171-20190) | ＜0.001 |
| Mean Pro-BNP | 4241(1419-8510) | 4022(1359-7948) | 6273(2026-11509) | ＜0.001 |
| Min HbA1c | 6.3(5.9-6.9) | 6.3(5.9-6.9) | 6.4(6.0-6.8) | 0.079 |
| Max HbA1c | 7.3(6.6-8.7) | 7.4(6.6-8.8) | 7.2(6.6-8.3) | 0.123 |
| Mean HbA1c | 6.8(6.2-7.7) | 6.8(6.2-7.7) | 6.8(6.3-7.6) | 0.714 |
| **Disease score** |  |  |  |  |
| APS III | 49(39-64) | 47(38-60) | 76(60-96) | ＜0.001 |
| SOFA | 5(3-7) | 5(3-7) | 8(6-12) | ＜0.001 |
| SIRS | 2(2-3) | 2(2-3) | 3(2-3) | ＜0.001 |
| **Comorbidity** |  |  |  |  |
| Hypertension(n(%)) | 1908(59.4) | 1693(60.1) | 215(54.4) | 0.033 |
| Arrhythmia(n(%)) | 1237(38.5) | 1091(38.8) | 146(37.0) | 0.838 |
| Cardiomyopathy(n(%)) | 755(23.5) | 660(23.4) | 95(24.1) | 0.800 |
| Coronary disease(n(%)) | 1938(60.4) | 1693(60.1) | 245(62.0) | 0.510 |
| MI(n(%)) | 941(29.3) | 817(29.0) | 124(31.4) | 0.345 |
| Peripheral vascular disease(n(%)) | 615(19.2) | 544(19.3) | 71(18.0) | 0.585 |
| Cerebral disease(n(%)) | 516(16.1) | 454(16.1) | 62(15.7) | 0.884 |
| Valvular disease(n(%)) | 705(22.0) | 625(22.2) | 80(20.3) | 0.399 |
| COPD(n(%)) | 487(15.2) | 422(15.0) | 65(16.5) | 0.454 |
| Respiratory failure(n(%)) | 799(24.9) | 699(24.8) | 100(25.3) | 0.852 |
| Pulmonary heart diseases(n(%)) | 792(24.7) | 690(24.5) | 102(25.8) | 0.575 |
| AKI(n(%)) | 1824(56.8) | 1591(56.5) | 233(59.0) | 0.357 |
| CKD(n(%)) | 1674(52.1) | 1459(51.8) | 215(54.4) | 0.361 |
| Hyperlipidemia(n(%)) | 2111(65.8) | 1860(66.1) | 251(63.5) | 0.336 |
| Hypothyroidism(n(%)) | 610(19.0) | 537(19.1) | 73(18.5) | 0.837 |
| Hemopathy(n(%)) | 852(26.5) | 735(26.5) | 107(27.1) | 0.808 |
| **Drug use** |  |  |  |  |
| Insulin(n(%)) | 981(30.6) | 849(30.2) | 132(33.4) | 0.199 |
| Loop diuretic(n(%)) | 2508(78.1) | 2198(78.1) | 310(78.5) | 0.897 |
| β-blocker(n(%)) | 2211(68.9) | 1934(68.7) | 277(70.1) | 0.602 |
| Digoxin(n(%)) | 270(8.4) | 235(8.3) | 35(8.9) | 0.699 |
| Albumin(n(%)) | 474(14.8) | 411(14.6) | 63(15.9) | 0.495 |
| Dobutamine(n(%)) | 115(3.6) | 103(3.7) | 12(3.0) | 0.664 |
| ACEI/ARB(n(%)) | 1180(36.8) | 1033(36.7) | 147(37.2) | 0.867 |
| Epinephrine(n(%)) | 214(6.7) | 188(6.7) | 26(6.6) | 1.000 |
| Norepinephrine(n(%)) | 732(22.8) | 649(23.1) | 83(21.0) | 0.405 |
| CCB(n(%)) | 532(16.6) | 461(16.4) | 71(18.0) | 0.427 |

**Supplemental Table 2 Baseline characteristics and clinical outcomes between patients treated with insulin medication and those without insulin after propensity score matching**

|  | Use(N=699) | Non-use(N=699) | P-Value |
| --- | --- | --- | --- |
| Age | 75(66-83) | 74(66-83) | 0.210 |
| Min Urine output | 25(10-50) | 25(10-50) | 0.810 |
| Max Urine output | 400(290-600) | 400(290-600) | 0.807 |
| Max Lactate | 3.2(2.1-4.5) | 3.2(2.1-4.6) | 0.989 |
| Min Lactate | 1.0(0.8-1.2) | 1.0(0.8-1.2) | 0.540 |
| Min bicarbonate | 19(16-22) | 19(16-21) | 0.407 |
| Max bicarbonate | 33(30-36) | 33(30-37) | 0.708 |
| Mean RR | 20(17-22) | 19(17-22) | 0.286 |
| Max HR | 98(85-113) | 98(85-112) | 0.725 |
| Max ALT | 46(26-133) | 49(26-152) | 0.453 |
| Min SBP | 90(81-101) | 90(80-101) | 0.914 |
| Mean SBP | 116(106-129) | 117(106-130) | 0.675 |
| Max Bilirubin | 0.8(0.5-1.6) | 0.9(0.5-1.7) | 0.662 |
| Max BUN | 63(40-93) | 62(41-95) | 0.562 |
| Min MBP | 56(49-63) | 56(49-63) | 0.946 |
| Max Pro-BNP | 7795(3248-16046) | 8074(3008-16559) | 0.708 |
| Min albumin | 3.0(2.5-3.4) | 3.0(2.5-3.4) | 0.942 |
| **Clinical outcomes** |  |  |  |
| SOFA | 7(5-10) | 8(5-11) | 0.743 |
| APS III | 63(49-84) | 65(49-86) | 0.683 |
| Hospital mortality | 86 | 85 | 1.000 |

**Supplemental Table 3 Baseline characteristics and clinical outcomes between patients treated with diuretics medication and those without diuretics after propensity score matching**

|  | Use(N=965) | Non-use(N=965) | P-Value |
| --- | --- | --- | --- |
| Age | 73(65-82) | 73(65-81) | 0.906 |
| Min Urine output | 25(10-50) | 25(10-50) | 0.592 |
| Max Urine output | 400(300-600) | 400(275-600) | 0.315 |
| Max Lactate | 3.2(2.2-4.8) | 3.2(2.2-4.8) | 0.354 |
| Min Lactate | 1.0(0.8-1.2) | 1.0(0.8-1.3) | 0.224 |
| Min bicarbonate | 18(15-21) | 18(15-21) | 0.212 |
| Max bicarbonate | 33(30-37) | 33(30-36) | 0.489 |
| Mean RR | 19(17-22) | 19(17-22) | 0.828 |
| Max HR | 97(85-112) | 98(86-110) | 0.328 |
| Max ALT | 47(26-138) | 51(28-157) | 0.430 |
| Min SBP | 90(80-101) | 90(81-101) | 0.911 |
| Mean SBP | 116(106-129) | 115(106-129) | 0.991 |
| Max Bilirubin | 0.9(0.5-1.8) | 0.9(0.6-1.7) | 0.780 |
| Max BUN | 66(43-99) | 65(41-98) | 0.972 |
| Min MBP | 56(50-63) | 56(50-64) | 0.577 |
| Max Pro-BNP | 8180(3367-16576) | 8202(3386-15999) | 0.826 |
| Min albumin | 3.0(2.5-3.4) | 3.0(2.5-3.4) | 0.807 |
| **Clinical outcomes** |  |  |  |
| SOFA | 5(3-8) | 5(3-7) | 0.360 |
| APS III | 50(39-64) | 49(39-64) | 0.518 |
| Hospital mortality | 128 | 108 | 0.187 |

**Supplemental Table 4 The number and proportion of missing values for each indicator**

|  | Number | Proportion |
| --- | --- | --- |
| Age (years） | 0 | 0 |
| Males(n(%)) | 0 | 0 |
| HR (/min) | 0 | 0 |
| RR(/min) | 0 | 0 |
| Temperature(℃) | 0 | 0 |
| SpO2(%) | 0 | 0 |
| SBP(mmHg) | 0 | 0 |
| DBP(mmHg) | 0 | 0 |
| MBP(mmHg) | 0 | 0 |
| **Lab events** | 0 | 0 |
| Glucose(mmol/L) | 0 | 0 |
| WBC (K/uL) | 0 | 0 |
| RBC(m/uL) | 914 | 0.284735 |
| Platelet(K/uL) | 917 | 0.28567 |
| Hemoglobin(g/dL) | 889 | 0.276947 |
| Anion gap(mEq/L) | 715 | 0.222741 |
| Bcarbonate(mEq/L) | 712 | 0.221807 |
| Sodium(mmol/L) | 607 | 0.189097 |
| Potassium(mmol/L) | 544 | 0.16947 |
| Chloride(mmol/L) | 620 | 0.193146 |
| BUN (mg/dL) | 514 | 0.160125 |
| Creatinine(mg/dL) | 420 | 0.130841 |
| Lactate(mg/dL) | 0 | 0 |
| Albumin(g/dL) | 219 | 0.068224 |
| ALT(IU/L) | 153 | 0.047664 |
| Bilirubin (mg/dL) | 0 | 0 |
| Urine output(ml) | 161 | 0.050155 |
| CK-MB(ng/mL) | 1571 | 0.489408 |
| Troponin-T(ng/mL) | 1458 | 0.454206 |
| NT-Pro-BNP(pg/mL) | 896 | 0.279128 |
| HbA1c(%) | 0 | 0 |
| **Disease score** |  |  |
| APS III | 0 | 0 |
| SOFA | 0 | 0 |
| SIRS | 0 | 0 |
